# The ratio of urinary TREM-1/TREM-2 mRNA expression in chronic kidney disease and renal fibrosis

**DOI:** 10.1080/07853890.2021.1912384

**Published:** 2021-06-28

**Authors:** Yuhan Cao, Yuwei Wang, Nana Peng, Jie Xiao, Sufen Wang, Cong Fu

**Affiliations:** a Department of Nephrology, Yi Ji Shan Hospital Affiliated to Wan Nan Medical College, China; b Key Laboratory of Non-coding RNA Transformation Research of Anhui Higher Education Institution (Wann Nan Medical College), China; c School of Clinical Medicine, Wan Nan Medical College, China; d School of Anesthesiology, Wan Nan Medical College, China; e Department of Pathology, Yi Ji Shan hospital affiliated to Wan Nan Medical College, China; f Department of Cardiology, Yi Ji Shan hospital affiliated to Wan Nan Medical College, China

**Keywords:** CKD, renal fibrosis, urinary mRNA, TREM-1, TREM-2, biomarker

## Abstract

**Background:**

The non-invasive identification of novel renal fibrosis biomarkers needs to be further studied.

**Methods:**

We collected urine samples from 77 biopsy-proven CKD patients and 15 healthy controls. The expression of urinary TREM-1 and TREM-2 was measured and the correlation with renal function parameter and pathological indicators was performed. The receiver operating characteristic (ROC) curve for the diagnosis of renal fibrosis was calculated. The protein expression of TREM-1 and TREM-2 in kidney tissues was measured.

**Results:**

The TREM-1/TREM-2 ratio was decreased in CKD patients and correlated with serum creatinine, estimated glomerular filtration rate and cystatin c. Further, the TREM-1/TREM-2 ratio was significantly decreased in moderate-severe fibrosis patients compared with none-mild renal fibrosis. TREM-1/TREM-2 ratio was correlated with the score of tubulointerstitial fibrosis (TIF) and the score of glomerular sclerosis. The ROC curve showed that the urinary TREM-1/TREM-2 ratio can diagnosemoderate-severe renal fibrosis at a cut-off value of 1.338 with a sensitivity of 86.4% and specificity of 81.8%. In human moderate-severe fibrosis kidney tissue, the protein expression of TREM-1 was lower and the TREM-2 was higher than none-mild fibrosis kidney tissue.

**Conclusion:**

Urinary TREM-1/TREM-2 ratio was a potential biomarker for the diagnosis of renal fibrosis in CKD patients.

## Introduction

Chronic kidney disease (CKD) is a major public health problem worldwide and in China. The recent cross-sectional survey focussed on Chinese people showed that the morbidity of CKD is 10.8% [1]. The end-stage renal disease is the common ending of CKD. Renal fibrosis which is characterized by glomerulosclerosis and tubulointerstitial fibrosis (TIF) is the common pathological findings of end-stage renal disease [2].

A diagnosis of renal fibrosis relies on renal biopsy and pathological staining. Although the severe complications of renal biopsy such as bleeding are rare in clinical practice, repeated renal biopsy is difficult to perform. So far, there is no appropriate method for dynamic monitoring of the progression of renal fibrosis [3]. It is important to have a non-invasive procedure for the identification of biomarkers that can predict and monitor the progression of renal fibrosis.

Real-time quantitative polymerase chain reaction (qPCR)-based urinary RNA detection has been developed for years and provided a novel strategy for the identification of renal kidney and CKD biomarkers [4]. The triggering receptor expressed on myeloid cells-1 (TREM-1) and triggering receptor expressed on myeloid cells-2 (TREM-2) is a cell surface receptor primarily expressed on monocyte-derived cells. In recent years, researchers found that TREM-1 participated in the progression of CKD and renal fibrosis [5,6]. Furthermore, previous studies also revealed the role of Trem-2 in the differentiation of inflammatory cells and immunomodulation, even in kidney disease [7]. In general, TREM-2 may participate in an assisted role, while the TREM-1 performed a biological effect. However, the role of TREM-2 in CKD and renal fibrosis has not been further studied. If the urinary mRNA expression of TREM-1 and TREM-2 have correlated with CKD and renal fibrosis was still unknown. Accordingly, this study was designed to determine the expression of urinary TREM-1 and TREM-2 *via* qPCR indicate CKD and renal fibrosis.

## Methods

### Study design and participants

**Figure 1.**
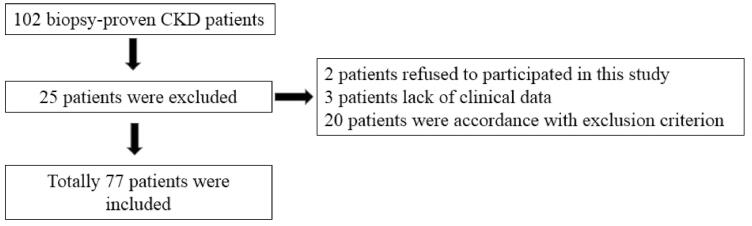
The flow diagram of this study.

A total of 77 biopsy-proven CKD patients were selected from the Department of Nephrology, Yi Ji Shan Hospital, Wannan Medical College from 2018–2019. The flow diagram for selecting patients was shown in Figure 1. The 77 patients containing IgA nephropathy (*n* = 38), membranous nephropathy (*n* = 8), minimal change disease (*n* = 6), focal segmental glomerulosclerosis (*n* = 7), diabetic nephropathy (*n* = 8), hypertensive nephropathy (*n* = 2) and non-IgA mesangioproliferative glomerulopathy (*n* = 8). The exclusion criteria were used: patients younger than 18 years old or older than 80 years old; patients with chronic liver disease, urinary tract infection, cancer, or organ transplantation; CKD patients with severe complications: cardiovascular disorder; or the use of steroids or immunosuppressive medications. The clinical data of all participants were collected. A total of age- and gender-matched healthy volunteers (*n* = 15) from the Yi Ji Shan Hospital Health Care Centre were also enrolled in the study as controls. Healthy controls were defined by the absence of abnormalities on a routine urinalysis and normal renal function [estimated glomerular filtration rate (eGFR)>90 ml^−1^·min^−1^·1.73 *m*^−2^].

### Collection of urine samples and RNA isolation

The whole stream early morning urine specimens were collected 24 h after renal biopsy. Urine samples were centrifuged at 3000 *g* for 30 min at 4 °C. The remaining cell pellets were collected and then resuspended in 1.5 ml DEPC-treated PBS and centrifuged at 13,000 *g* for 5 min at 4 °C. After being washed three times by diethylpyrocarbonate- (DEPC-) treated phosphate buffer saline (PBS), the pellets were resuspended in 1.0 ml TRIzol Reagent (Ambion, Life Technologies) and stored at −80 °C. Total RNA was extracted according to the manufacturer’s protocol (Ambion, Life Technologies, USA). Furthermore, the concentration and purity of RNA were assessed using the relative absorbance ratio at 260/280 in a NanoDrop 2000 (Thermo, USA). GAPDH mRNA was measured as the control.

### Real-Time RT-qPCR

RT-PCR was performed using TREM-1 primers (sense: 5’AGTTACAGCCCAAAACATGC-3′; antisense: 5′-CAGCCCCCACAAGAGAATTA-3′), TREM-2 primers (sense: 5′-ACAGAAGCCAGGGACACATC-3′; antisense: 5′-CCTCCCATCATCTTCCTTCA-3′) and GAPDH primers (sense: 5′-GGTGAAGGTCGGAGTCAACGGATTTGGTCG-3′; antisense: 5′-GGATCTCGCTCCTGGAAGATGGTGATGGG-3′). After RT (50 °C, 30 min), hot start (94 °C, 15 min), and 40–42 cycles of PCR (94 °C, 1 min; 52.5 °C, 1 min; and 72 °C, 1 min), TREM-1 and TREM-2 mRNA expression was normalised to GAPDH and calculated as 2^−ΔΔCt^.

### Renal fibrosis

Renal fibrosis was measured on paraffin-embedded sections stained with Masson trichrome. Serial 3 μm sections were acquired from each paraffin block. Two experienced pathologists who were blinded to the results of molecular studies subjectively scored the severity of renal fibrosis. Glomerulosclerosis was assessed in periodic acid-Schiff-stained sections using a semiquantitative scoring system according to the method of Schaier et al. [8]. Each glomerulus was graded from 0 to 4 according to sclerosis severity, and the average of all glomeruli in the entire tissue sample was calculated for analysis. The evaluation of the percentage of TIF was performed on Masson-stained sections and estimated the severity of TIF [9]. None-mild was considered to be <25% of the renal interstitium. Moderate-severe was considered to be ≥25%.

### Immunofluorescence

Kidney tissue sections were deparaffinized in xylene and rehydrated in a graded series of ethanol. The section (2 μm thick) was stained with goat anti-human TREM-2 antibody (R&D system, USA) and rabbit anti-Human TREM-1 antibody (LSBio, USA). The secondary antibody was Alexa Fluor594 labelled donkey anti-rabbit antibody (Abcam, UK) and Cy-3 labelled donkey anti-goat antibody (Beyotime, China). Fluorescence microscopy (Olympus, Tokyo, Japan) was used to detect the fluorescence.

### Statistical analysis

SPSS 17.0 was used for data analysis. Relative changes in gene expression were calculated using the ΔΔCt (threshold cycle) method: ΔCt = Ct gene of interest‐Ct internal control, while ΔΔCt = Ct gene of interest‐Ct internal control sample‐ Ct gene of interest‐Ct internal control. Fold change = target gene expression level of sample/target gene expression level of control = 2 ^− ΔΔCt^. Normal distribution and equal variance data were expressed as mean ± standard deviation and compared using Student’s t-test. Variance inequality or non-normal distribution data were expressed as median (min, max). A Mann–Whitney test was used for variance inequality or non-normal distribution data. Spearman’s rank-order correlation coefficient was used to assess associations between gene expression levels and clinical parameters. Stepwise multivariate logistic regression analysis was used to assess the predictors for renal fibrosis. The diagnostic performance of biomarkers was evaluated using receiver operating characteristic (ROC) curves. The diagnostic threshold for maximum sensitivity and specificity was calculated. All P values were two-tailed, and *p* < .05 was considered statistically significant.

## Results

### Baseline characteristic

**Figure 2.**
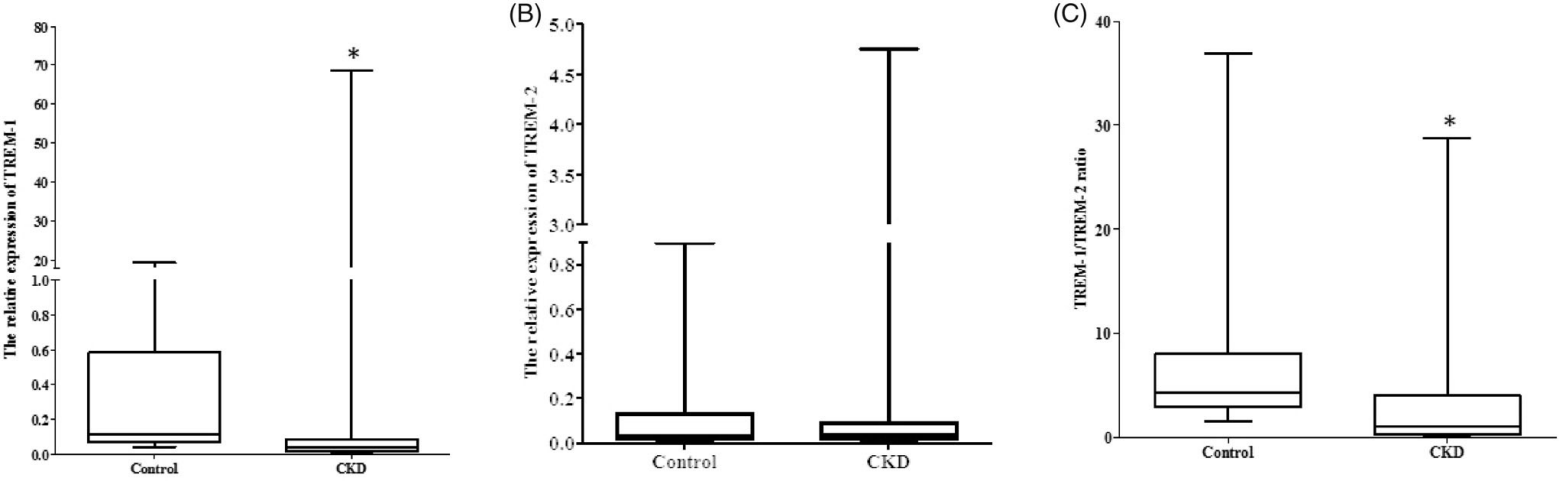
Urinary TREM-1 and TREM-2 mRNA expression in CKD patients and controls. (A) The relative expression of urinary TREM-1 in CKD and health control. (B) The relative expression of urinary TREM-2 in CKD and health control. (C) The TREM-1/TREM-2 ratio in CKD and health control (*p < .05 vs control).

The primary clinical and pathological characteristics of the involved subjects are shown in Table 1. There were no significant differences in age and gender between CKD patients and controls. The CKD group had a significant decrease in the estimated glomerular filtration rate (eGFR) compared with controls. eGFR was calculated using modified MDRD equations for Chinese patients [18]. Relative expression of TREM-1 was significantly decreased in the CKD group (*p* < .001 vs control, Figure 2(A)). The expression of TREM-2 was higher in the CKD group. However, no statistical differences were observed (*p* = .937 vs control, Figure 2(B)). The TREM-1/TREM-2 ratio was significantly lower in the CKD group (*p* = .001 vs control, Figure 2(C)).

**Table 1.** Clinical profile of patients with CKD and healthy controls at the time of kidney biopsy.

	CKD (*n* = 77)	Control (*n* = 15)	*p* Value
Age (years)	45.9 ± 14.6	38.8 ± 7.0	.070
Gender (male/female)	38/39	9/6	.450
24h Proteinuria (g/day)	1.710 (0.020–9.760)	/	/
Scr (mmol/l)	100.2 ± 74.1	60.3 ± 6.7	.041
BUN (mmol/L)	7.1 ± 4.0	4.0 ± 0.8	.004
Cystatin C (mg/L)	1.36 ± 0.82	0.67 ± 0.15	.002
eGFR(ml/min per 1.73 m^2^)	85.2 ± 32.8	127.9 ± 18.1	<.001
SBP (mmHg)	139.0 ± 22.2	122.2 ± 6.2	<.001
DBP (mmHg)	85.1 ± 14.0	75.0 ± 5.6	.006
Relative TREM-1 expression	0.037 (0.001–68.696)	0.110 (0.041–19.252)	<.001
Relative TREM-2 expression	0.041 (0.001–4.457)	0.033 (0.005–0.898)	.937
TREM-1/TREM-2 Ratio	1.007 (0.005–28.742)	4.324 (1.506–36.928)	.001
Score of glomerular sclerosis	1.45 (0.62–4.00)	/	/
Score of TIF, %	30.0 (0.0–90.0)	/	/

Scr: serum creatinine; eGFR: estimated glomerular filtration rate; BUN: blood urea nitrogen; SBP: systolic blood pressure; DBP: diastolic blood pressure.

**Figure 3.**
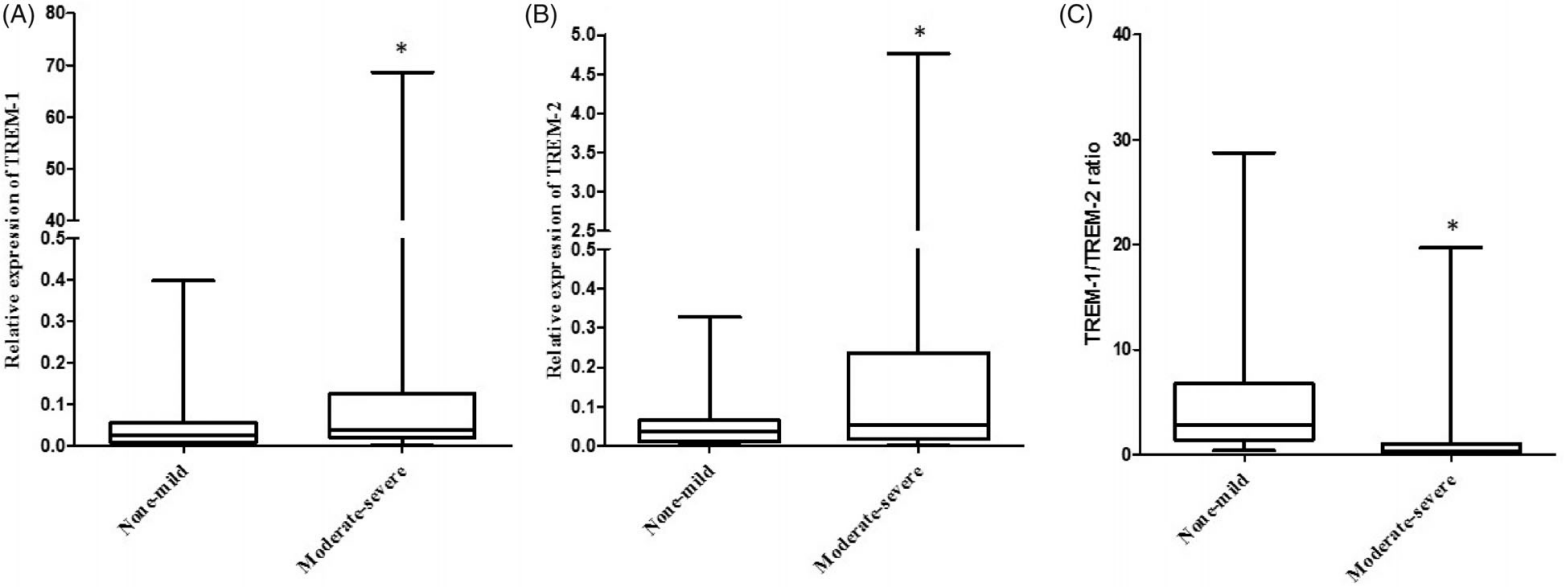
Urinary TREM-1 and TREM-2 mRNA expression in different fibrosis degree in CKD patients. (A) The relative expression of urinary TREM-1 in different degrees of renal fibrosis patients. (B) The relative expression of urinary TREM-1 in different degrees of renal fibrosis patients. (C) The TREM-1/TREM-2 ratio in different degrees of renal fibrosis patients (*p < .05 vs none-mild).

**Figure 4.**
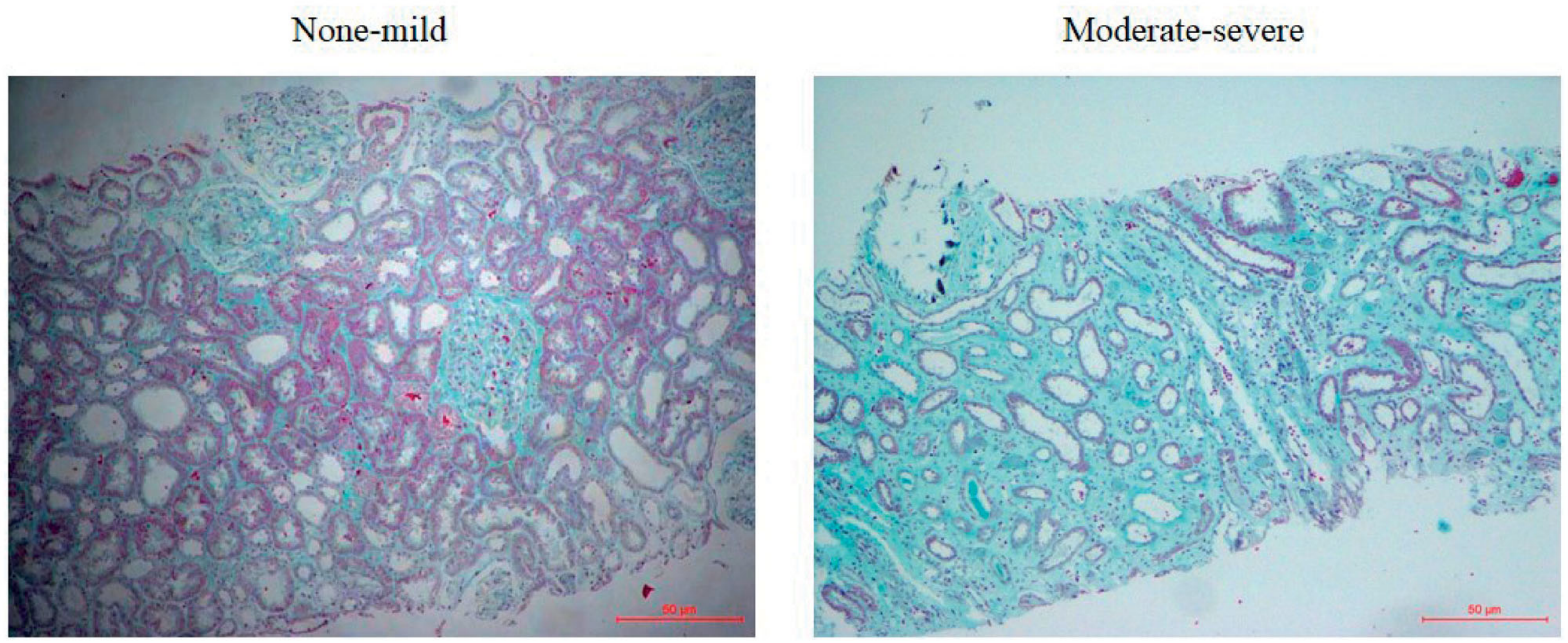
Representative histological findings of renal fibrosis stained by Masson’s trichrome. None-to-mild fibrosis was considered as <25% of the tubulointerstitial fibrosis (TIF) area, moderate-severe fibrosis referred to an area ≥25% of the TIF area. Original magnification: *100.

The 77 CKD patients were divided into two groups according to renal fibrosis degree. As shown in Table 2, there were no significant differences in age, gender, 24-h proteinuria, Scr, Cystatin C and BUN between the two groups. The eGFR in moderate-severe renal fibrosis was significantly lower compared with the none-mild group. The relative expression of TREM-1 (*p* = .020 vs none-mild, Figure 3(A)) and TREM-2 (*p* = .033 vs none-mild, Figure 3(B)) was significantly higher in the moderate-severe group. The TREM-1/TREM-2 ratio was significantly lower in the moderate-severe group (*p* < .001 vs none-mild, Figure 3(C)). Figure 4 showed the representative of different degrees of renal fibrosis confirmed by Masson trichrome.

**Table 2.** Clinical and pathological parameter of patients with renal fibrosis.

	None-mild fibrosis (*n* = 33)	Moderate-Severe fibrosis (*n* = 44)	*p* Value
Age (years)	45.5 ± 15.2	46.1 ± 14.4	.862
Gender (male/female)	14/19	24/20	.359
24h Proteinuria (g/day)	1.560 (0.020–9.160)	1.925 (0.020–9.760)	.362
Scr (μmol/l)	88.1 ± 61.2	116.5 ± 86.9	.096
BUN (mmol/L)	6.5 ± 3.6	8.0 ± 4.5	.102
Cystatin C (mg/L)	1.22 ± 0.69	1.56 ± 0.94	.075
eGFR (ml/min per 1.73 m^2^)	92.3 ± 29.6	75.8 ± 34.9	.028
SBP (mmHg)	128.9 ± 23.1	131.5 ± 21.5	.616
DBP (mmHg)	84.2 ± 11.6	87.1 ± 16.6	.360
Relative TREM-1 mRNA expression	0.027 (0.002–0.399)	0.039 (0.001–68.696)	.020
Relative TREM-2 mRNA expression	0.037 (0.001–0.327)	0.052 (0.003–4.757)	.033
TREM-1/TREM-2 Ratio	2.792 (0.352–28.742)	0.334 (0.005–19.685)	<.001
Score of glomerular sclerosis	1.11 (1.00–2.41)	2.00 (0.62–4.00)	.053
Score of TIF (%)	12.00 (0.00–24.00)	41.00 (27.00–90.00)	<0.001

Scr: serum creatinine; eGFR: estimated glomerular filtration rate; BUN: blood urea nitrogen; SBP: systolic blood pressure; DBP: diastolic blood pressure.

### Correlation between urinary TREM-1/TREM-2 ratio, renal function parameters and fibrosis

**Figure 5.**
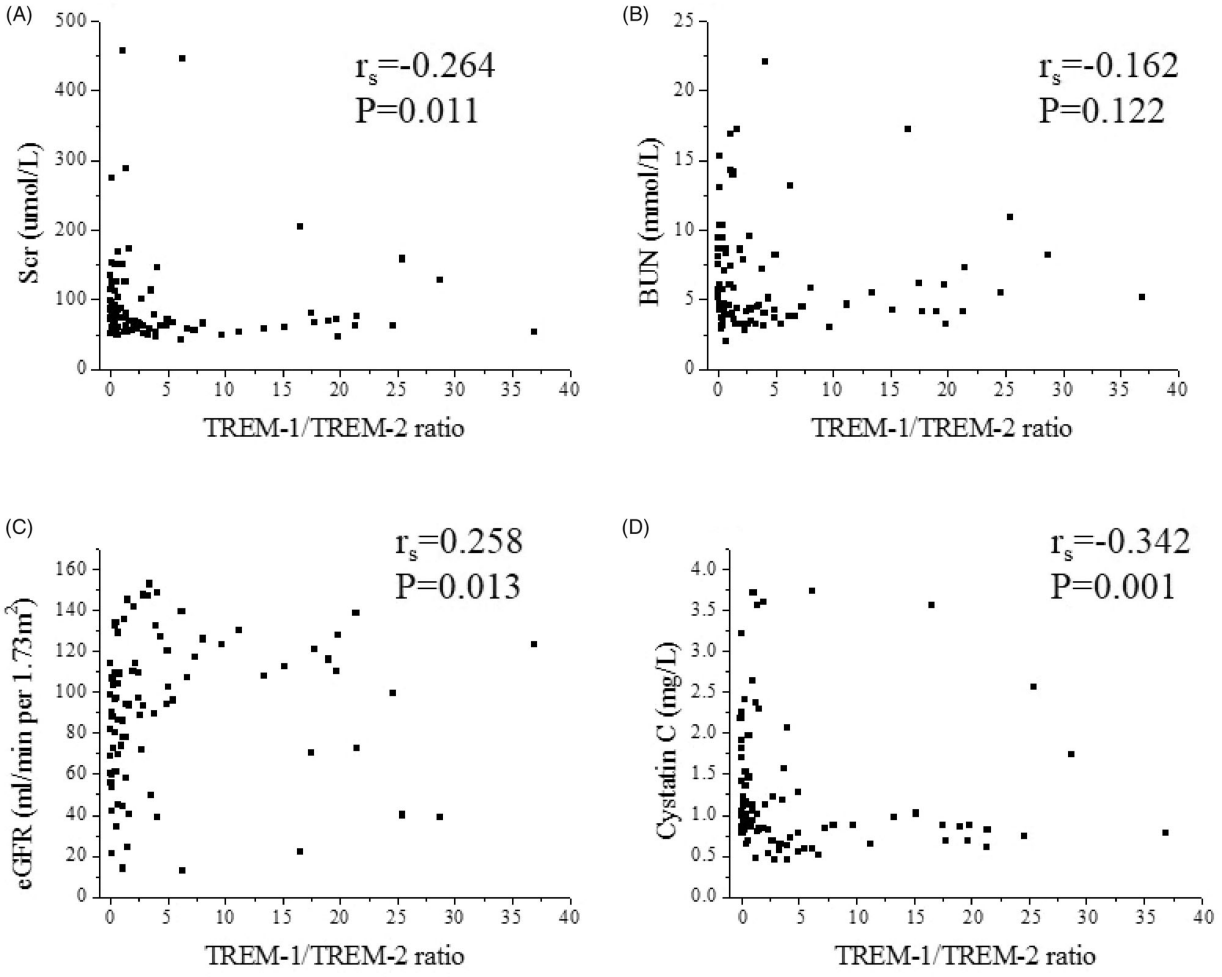
Correlation between urinary TREM-1/TREM-2 ratio and clinical parameters. (A) Spearman correlation between TREM-1/TREM-2 ratio and Scr (r_s_= −0.264, *p* = .011). (B) Spearman correlation between TREM-1/TREM-2 ratio and BUN (r_s_= −0.162, *p* = .122). (C) Spearman correlation between TREM-1/TREM-2 ratio and eGFR (r_s_=0.258, *p* = .013). (D) Spearman correlation between TREM-1/TREM-2 ratio and cystatin c (r_s_= −0.342, *p* = .001).

**Figure 6.**
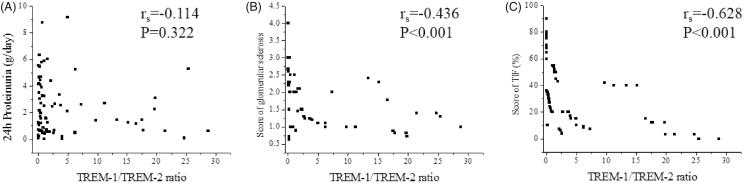
Correlation between urinary TREM-1/TREM-2 ratio and 24 h Proteinuria, renal fibrosis. (A) Spearman correlation between urinary TREM-1/TREM-2 ratio and 24-h Proteinuria (r_s_=-0.114, *p* = .322). (B) Spearman correlation between urinary TREM-1/TREM-2 ratio and score of glomerular sclerosis (r_s_= −0.436, *p* < .001). (C) Spearman correlation between urinary TREM-1/TREM-2 ratio and score of TIF (r_s_= −0.628, *p* < .001).

Urinary TREM-1/TREM-2 ratio correlated with Scr (r_s_=−0.264, *p* = .011), eGFR (r_s_=0.258, *p* = .013) and Cys-c (r_s_= −0.342, *p* = .001). However, there was no correlation between TREM-1/TREM-2 and BUN (r_s_= −0.162, *p* = .122) (Figure 5). Furthermore, in CKD patients, urinary TREM-1/TREM-2 ratio correlated with score of glomerular sclerosis (rs= −0.436, *p* < .001) and score of TIF (rs= −0.628, *p* < .001). However, there was no correlation between TREM-1/TREM-2 and 24 h Proteinuria (r_s_= −0.114, *p* = .322) (Figure 6).

Stepwise multivariate logistic regression analysis further showed that the relative expression of urinary TREM-1/TREM-2 ratio significantly correlated with the severity of renal fibrosis (Table 3, OR, 0.909, 95% CI, 0.839–0.985, *p* = .020). The results indicated that the urinary TREM-1/TREM-2 ratio decreased every one unit, the risk for renal fibrosis elevated by 9.1%.

**Table 3.** Multivariate logistic regression analysis of selected variables for renal fibrosis severity.

	OR	95% CI	*p* Value
TREM-1/TREM2 ratio	0.909	0.839–0.985	.020
Scr	1.003	0.990–1.016	.662
BUN	1.043	0.848–1.282	.692
Cystatin C	0.664	0.195–2.264	.513
eGFR	1.023	0.995–1.053	.113
24h proteinuria	1.149	0.888–1.486	.290

Scr: serum creatinine; eGFR: estimated glomerular filtration rate; BUN: blood urea nitrogen; OR: odds ratio; CI: confidence interval.

### Diagnostic value of urinary TREM-1/TREM-2 ratio

**Figure 7.**
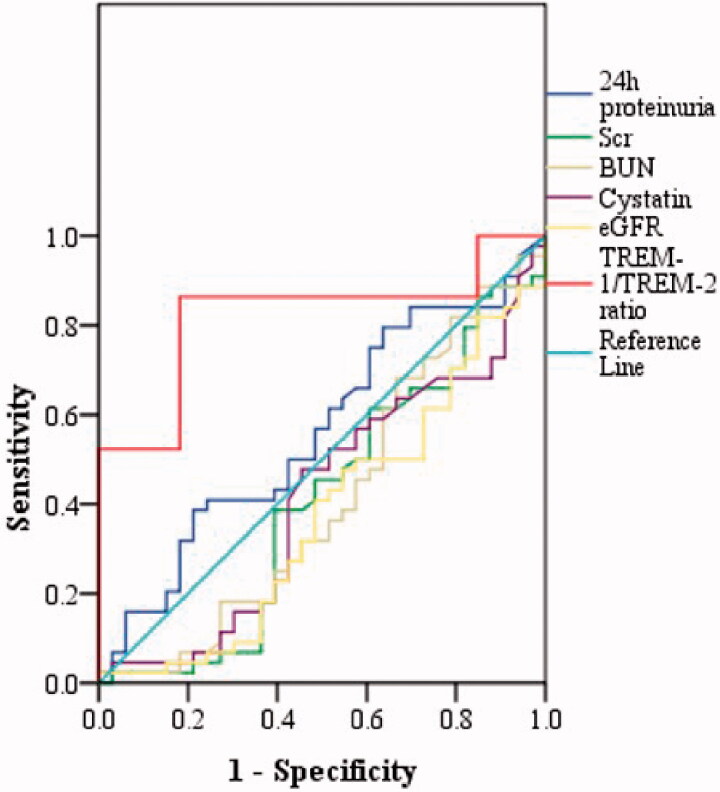
The receiver operating characteristic (ROC) curve showed the diagnosis value of the urinary TREM-1/TREM-2 ratio for renal fibrosis. ROC curve showed the urinary TREM-1/TREM-2 ratio distinguished moderate-to-severe fibrosis from none-mild fibrosis (AUC of 0.822; 95% CI, 0.725–0.920; *p* < .001).

We evaluated the diagnostic value of urinary TREM-1/TREM-2 ratio for renal fibrosis. The results showed that urinary TREM-1/TREM-2 ratio effectively distinguished moderate-to-severe fibrosis from none-mild fibrosis, with the largest AUC of 0.822 (95% CI, 0.725–0.920; *p* < .001) higher than that of eGFR, (AUC of 0.373; 95% CI, 0.245–0.500; *p* = .057), Scr (AUC of 0.407; 95% CI, 0.275–0.540; *p* = .166), BUN (AUC of 0.411; 95% CI, 0.278–0.544; *p* = .184), 24h proteinuria (AUC of 0.561; 95% CI, 0.431–0.691; *p* = .362) and Cys-c (AUC of 0.413; 95% CI, 0.282–0.543; *p* = .191). UrinaryTREM-1/TREM-2 ratio displayed the sensitivity of 86.4% and specificity of 81.8% at the optimal cut-off value of 1.338 (Figure 7).

### The expression of TREM-1 and TREM-2 expression in kidney tissue

**Figure 8.**
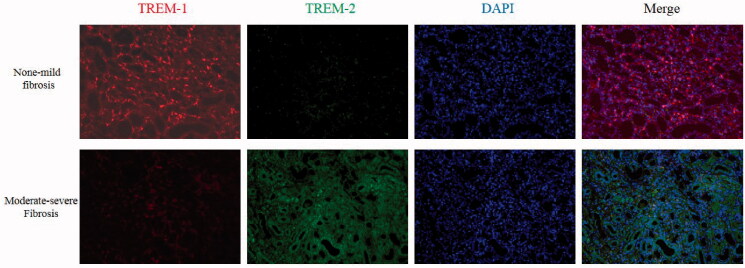
The expression of TREM-1 and TREM-2 expression in kidney tissue. First line: In none-mild fibrosis kidney tissue, the TREM-1 protein expression was significantly higher than TREM-2 expression. Second line: In moderate-severe fibrosis kidney tissue, the TREM-2 protein expression was significantly higher than TREM-1 expression (Red: TREM-1 expression; Green: TREM-2 expression; Blue: DAPI showed the nucleus; original magnification: *200).

The Immunofluorescence showed that in none-mild fibrosis kidney tissue, the TREM-1 protein expression was significantly higher than TREM-2 expression. However, in moderated-severe fibrosis kidney tissue, the TREM-2 protein expression was significantly higher than TREM-1 expression (Figure 8).

## Discussion

Our study firstly indicated that the mRNA ratio of urinary TREM-1/TREM-2 was related to CKD and may serve as a potential non-invasive biomarker of renal fibrosis. So far, a renal biopsy was also a unique method that can give an accurate diagnosis of the pathological type of CKD and the degree of fibrosis. Although repeated biopsy can dynamically detect the progression of CKD and fibrosis, it is difficult for applied in clinical. A non-invasive method that can replace renal biopsy has great significance for the diagnosis and treatment of CKD.

The urine contained massive biological information including protein and RNA that can indicate the state of CKD and degree of renal fibrosis [10,11]. qPCR has been suggested as a reliable method to detect urinary renal biomarkers. In 2001, Li et al. first established a non-invasive approach to diagnose acute renal rejection of allografts by isolating and quantifying RNA of specific genes in urine cells [4]. Urinary sediment has attracted researchers' focus and has become an attractive resource for detecting the biomarker for kidney diseases [10–13]. Urinary mRNA expression also showed the value in diagnosing early renal fibrosis [14]. Our research revealed that urinary mRNA detection *via* qPCR was a credible method to find novel biomarkers for renal fibrosis. Urinary sediment was an appropriate source for renal fibrosis diagnosis.

Numbers of previous researches have revealed the TREM-1 played a key role in CKD [5,15]. Lo TH et al. found that in the ureteral obstruction mouse model, TREM-1 regulated the polarization of macrophages [6]. Du et al. further indicated that TREM-1 has the therapeutic value in anti-GBM-induced nephritis [16]. The polarization of macrophages played a vital role in renal fibrosis [17]. TREM-2 was considered as a protecting molecular that regulate the immune reaction [18,19]. There was no in-depth study on the relation between TREM-2 and kidney disease. In other organ injury and disease, the TREM-2 showed a significant organ protecting effect [20,21]. The TREM-1/TREM-2 ratio that expression on the surface of blood monocytes could help predict prognosis in patients with gliomas [22]. A previous study also suggested that the balance of TREM-1 and TREM-2 regulated the progression of acute lung injury [23]. In renal fibrosis, the expression of TREM-2 in kidney tissue may have differences compared to healthy control. In our data, we found that in none-mild renal fibrosis human kidney tissue, the expression of TREM-1 was higher than TREM-2. However, in moderated-severe fibrosis, the expression of TREM-1 and TREM-2 in kidney tissue was opposite to none-mild fibrosis. The TREM-1 and TREM-2 expression information contained in urinary sediment may be played as biomarkers of renal fibrosis. Our data also showed that the TREM-1/TREM-2 ratio in urinary sediment has correlated with renal function and fibrosis and has diagnosis value in renal fibrosis.

In summary, our study demonstrated that the urinary TREM-1/TREM-2 ratio could well predict renal fibrosis severity in CKD, which suggested this will serve as a novel independent non-invasive biomarker to monitor the progression of kidney fibrosis in CKD.

Our study also has some limitations. Firstly, the current study is a discovery study focussed on CKD, which contained multiple pathological types. If urinary TREM-1/TREM-2 ratio can better serve as a biomarker of renal fibrosis in specific pathological types need to be further studied. Secondly, this study did not separate the kidney cells from sediment. The urethral epithelial cells in urinary sediment may influence the reliability of this method. Thirdly, a large sample study and a long-term follow-up study are needed to further identify the value of urinary TREM-1 and TREM-2 in the diagnosis of CKD and renal fibrosis.

## Conclusion

Urinary TREM-1 and TREM-2 mRNA detection and the TREM-1/TREM-2 ratio served as a non-invasive biomarker of renal fibrosis.

## Data Availability

All data used during the study appeared in the submitted article
